# Effects of Processing Parameters on Surface Roughness of Additive Manufactured Ti-6Al-4V via Electron Beam Melting

**DOI:** 10.3390/ma10101121

**Published:** 2017-09-22

**Authors:** Pan Wang, Wai Jack Sin, Mui Ling Sharon Nai, Jun Wei

**Affiliations:** Forming Technology Group, Singapore Institute of Manufacturing Technology, 73 Nanyang Drive, Singapore 637662, Singapore

**Keywords:** 3D printing, surface roughness, processing parameter, multispot contouring, non-multispot contouring

## Abstract

As one of the powder bed fusion additive manufacturing technologies, electron beam melting (EBM) is gaining more and more attention due to its near-net-shape production capacity with low residual stress and good mechanical properties. These characteristics also allow EBM built parts to be used as produced without post-processing. However, the as-built rough surface introduces a detrimental influence on the mechanical properties of metallic alloys. Thereafter, understanding the effects of processing parameters on the part’s surface roughness, in turn, becomes critical. This paper has focused on varying the processing parameters of two types of contouring scanning strategies namely, multispot and non-multispot, in EBM. The results suggest that the beam current and speed function are the most significant processing parameters for non-multispot contouring scanning strategy. While for multispot contouring scanning strategy, the number of spots, spot time, and spot overlap have greater effects than focus offset and beam current. The improved surface roughness has been obtained in both contouring scanning strategies. Furthermore, non-multispot contouring scanning strategy gives a lower surface roughness value and poorer geometrical accuracy than the multispot counterpart under the optimized conditions. These findings could be used as a guideline for selecting the contouring type used for specific industrial parts that are built using EBM.

## 1. Introduction

Additive manufacturing (AM) has become a technique that global manufacturing industries are looking into in view of its capability to produce complex components without the need of assembly, surpassing that of conventional technologies. Electron beam melting (EBM) technology, as one of the layer-by-layer AM techniques, is introduced by Arcam AB, Sween. In the EBM system, high energy density electrons are generated to melt the metallic powder which enables it to process high melting point material. At the same time, the vacuum chamber environment prohibits the disturbance of oxygen, nitrogen, etc. while processing the reactive or sensitive materials such as titanium. In addition, a high preheat temperature up to 1100 °C significantly reduces the residual stress, which helps to reduce post heat treatments and support structures required during printing [1]. These advantages result in EBM being suitable for printing metal components with complex geometry and high accuracy, which is one of the main attractions of the EBM technology [2,3,4,5,6,7]. As a mature material, Ti-6Al-4V, has been developed by Arcam AB since 2004, with expected biomedical applications. Since then, the microstructure of EBM-built Ti-6Al-4V parts has been widely discussed [2,8,9,10,11,12,13,14] and the mechanical properties of a nearly full density Ti-6Al-4V part fabricated by EBM are strongly related to its microstructure and they distribute in a wide range [13,15,16,17,18]. Furthermore, the optimized EBM processing parameters result in as-built parts with comparable, if not better, mechanical properties than their wrought form [5,14,16,19,20]. These further make EBM-built Ti-6Al-4V components applicable in a lot of applications, such as biomedical implants, design, marine, and aerospace fields [4,5,8,21,22].

Due to the nature of the EBM building process, the as-built parts have relatively rough surfaces as compared to those conventionally machined surfaces [4,5,23,24,25]. The EBM part’s rough surface reduces the effective cross section [24,26], which changes the mechanical response [3,27] and results in premature failure of the part [24,28]. This is one of the reasons limiting the wide adoption of the EBM technology in the manufacture of parts for industrial use [29,30]. The partially melted powder (in terms of powder size), staircase effect (in terms of layer thickness), and process parameters are three key factors to influence the surface quality of the EBM components [3,23,24,25,29]. The metal powders typically used for the EBM technology range from 45 to 150 µm, the staircase effect is dependent on the curvature of the part’s surface and the deposition layer thickness that normally ranges from 50 to 200 µm. For a given powder size of Ti-6Al-4V (45–106 µm), the minimum layer thickness is almost fixed (50 µm) to achieve a better surface finish. In this case, the arithmetic roughness (Ra) of a typical EBM-built part, obtained from experimental results using the default parameters, ranged from 30 to 36 µm [5,25]. Hence, achieving an improved surface finish is essential. Although some post treatments—such as adaptive computer numerical control abrasive (grinding and/or polishing) material removal process, and chemical and plasma material removal process—can be applied to improve the surface roughness, the consideration of post treatments neutralizes the advantage of design freedom of EBM-built components. Further optimization of the EBM process parameters is thus of paramount importance to achieve a better surface finish. To date, there is limited study reported [29]. Here, an attempt is made to systemically understand the effect of processing parameters on the as-built part’s surface roughness. Furthermore, the judicious design of processing parameters is carried out to achieve the possible optimized parameters with improved surface roughness. Our results bridge the gap of limited understanding of surface roughness and could act as not only a database for simulation but also a selection criterion for industrial applications.

## 2. Experimental Procedure

**Figure 1:**
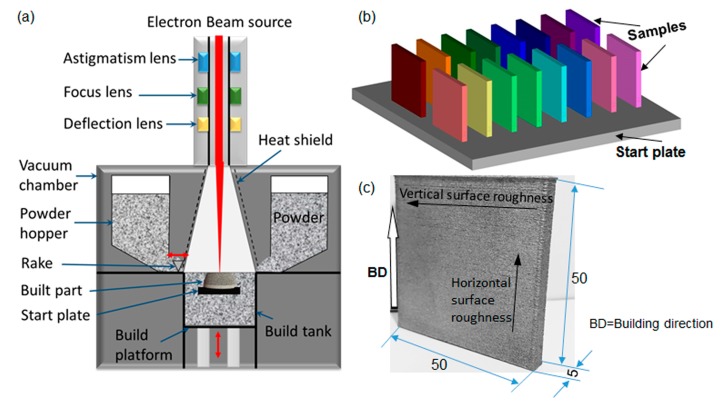
Illustration of (**a**) EBM system; (**b**) sample locations (each color indicated one sample); and (**c**) the picture of the EBM-built sample with dimensions (in mm). The thick arrow indicated the build direction and the thin arrows indicated the directions of the surface roughness measurements.

The samples analyzed in this study were fabricated using the Arcam A2X machine (Arcam AB, Mölndal, Sweden, illustrated in Figure 1a), with a fixed layer thickness of 50 µm. Ti-6Al-4V powder in its pre-alloyed form with a size range of 45–106 µm was provided by the EBM system manufacturer, Arcam AB (Mölndal, Sweden, software version 3.2). A detailed description of EBM process can be found elsewhere [4]. For each layer, the powder was raked and deposited over the build area and subsequently preheated to an elevated temperature followed by melting of the part. Both the preheating and melting processes were achieved by the energy transfer from a high-energy electron beam onto the powder bed. In the melting stage, the part is built in two steps. Firstly, the outer part or boundary is melted and referred to as the ‘contouring’. The contouring provides an interface between the actual build and the surrounding powder. The center of each section is then filled by rastering the beam in a snaking melting strategy which is known as ‘hatching’. Therefore, the contouring controls the surface roughness of the built parts. In the present study, the preheating and the hatching processes were kept at the default settings in order to minimize their influences.

**Figure 2:**
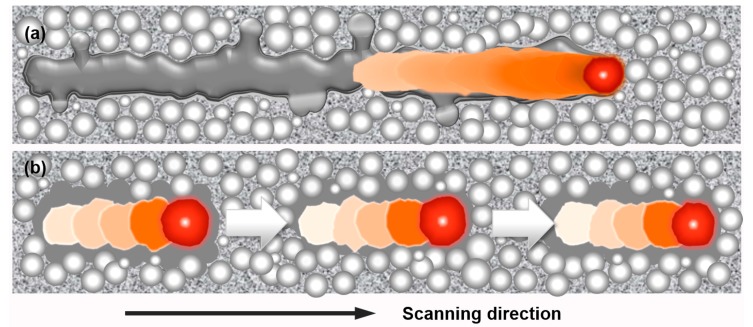
Illustration of (**a**) non-mulitspot and (**b**) mulitspot contouring strategies.

There are two types of contouring strategies in EBM processing, namely, (i) non-multispot contouring and (ii) multispot contouring which is also known as ‘MultiBeam’. The non-multispot contouring is a continuous translating melt process. Non-multispot contouring is achieved by the continuous electron beam melting the outer edges of the part at the specific layer of the build, as illustrated in Figure 2a. The scanning speed of the beam during contouring is calculated based on a built-in algorithm and is controlled by the speed function. For this method, the variable parameters are the contouring beam current (in mA), focus offset of the beam (in mA), and the speed function of the scanning. The multispot contouring which rapidly moves the beam so as to keep several separate melt pools active at one time is faster than the non-multispot contouring because of the lack of translation movement of the beam, as illustrated in Figure 2b. Multispot contouring is achieved by splitting up the contouring of the layer into shorter sections and for each section, the beam will ‘spot’ the outer edges of the part, almost simultaneously at multiple sections by moving to a subsequence section after each spot. Spotting is the melting of the powder in a region as small as the beam and over a short period of time. For this method, the variable parameters are the number of spots, spot time (in ms), the overlap of the spots (in mm), the contouring beam current (in mA), and the focus offset of the beam (in mA).

To exclude the location dependent effects (if any), five samples with the default processing parameters that located in the four corners and the center were firstly fabricated and tested. No location dependence effect was observed. Therefore, the sample located in the start plate can be considered as same conditions. In order to systematically examine the effect of the process parameters on the surface roughness, the sample was fabricated on a 210 mm square 10 mm thick stainless steel start plate, as shown in Figure 1b. Each color indicated one set of processing parameters. The sample had a cross-section of 5 × 50 mm^2^ and build height of 50 mm. The set of default processing parameters was applied in one of each batch samples to trace possible variation between batches. Only one sample was fabricated for the other sets of processing parameters. After optimization, some selected conditions, such as worst conditions, default conditions and best conditions, were fabricated to confirm the results. These processing parameters utilized in the present study were listed in Table 1 and Table 2 for non-multispot and multisport, respectively. Hereafter, the sample for non-multispot and multispot are designated as N and M, respectively. For example, sample 6 with non-mulispot scanning strategy was referred to as ‘N6’. During melting, the samples were arranged with 20 mm between each model, to minimize the thermal interaction. The stainless steel start plate was heated when the pressures of both the build chamber and electron beam column were below ~5 × 10^−4^ and ~5 × 10^−6^ mBar, respectively. Once the bottom temperature reached 730 °C, the parts were built directly onto the preheated start plate with a layer thickness of 50 µm by selective electron beam melting. The whole process was carried out under a vacuum of ~2 × 10^−3^ mBar which was controlled by using high purity helium as a regulating gas to prevent powder charging. During the process, the bottom temperature was kept at 620 to 650 °C. After finishing the build job and cooling to below 100 °C, a powder recovery system was applied to remove the surrounding semi-sintered power from the built parts and to recycle the used powders.

Following the manufacturing, the surface roughness measurements were carried out by using Accretech Surfcom FLEX stylus profilometer (Tokyo Seimitsu Co. Ltd., Tokyo, Japan) in both the parallel and perpendicular directions, with respect to the build direction, to compare the results among the various experiments, as illustrated in Figure 1c. Hereafter, we named the measured surface roughness in parallel to build direction as the vertical surface roughness and the measured surface roughness perpendicular to the build direction as the horizontal surface roughness. In accordance with ISO standard, ISO 4287-1:1997, the evaluation and cut-off lengths for all roughness measurements would be 40 mm and 8 mm, respectively. The measurements were recorded by having a diamond-tipped, spring-loaded cantilever stylus contact the surface of the samples and travel along the direction of interest. As it travels along the surface, the stylus will conform to the surface profile. The profilometer will track and record the stylus movement and calculate the roughness values accordingly. For each measurement direction, at least five measurements at various locations were made to obtain the average roughness values for all the samples in order to eliminate any discrepancy which resulted from surface irregularities. Moreover, some selected samples were characterized using an Alicona infinite focus microscope (IFM; IF-EdgeMaster G4 Vb, Alicona Imaging, Graz, Austria) to visualize the surface morphology and an optical microscope to evaluate the fusion conditions.

## 3. Results

### 3.1. Non-Multispot Contouring

Table 1 shows the measured surface roughness values, Ra in µm, in both vertical and horizontal directions for the non-multispot samples. It is revealed that the surface roughness of EBM-built sample can be varied by modifying the processing parameters. The lowest surface roughness is ~20 µm, compared to the highest surface roughness is ~40 µm, which differs from the previous reports by using an earlier version of EBM system, S12 [23,29]. Two optimized processing parameters are N5 and N6, as listed in Table 1. It is found that both the speed function and beam current have a significant effect on surface roughness. The minimum surface roughness value appeared in the mid-point of the experimental values for both the speed function and beam current. On the other hand, focus offset during non-multispot contouring had limited effect on the experimental results, which can be revealed by observing the trend of the experiments with the same beam current and speed function but different focus offsets. The results show that the difference between the pairs of experiments was insignificant to determine the effects of focus offset on the surface roughness.

**Figure 3:**
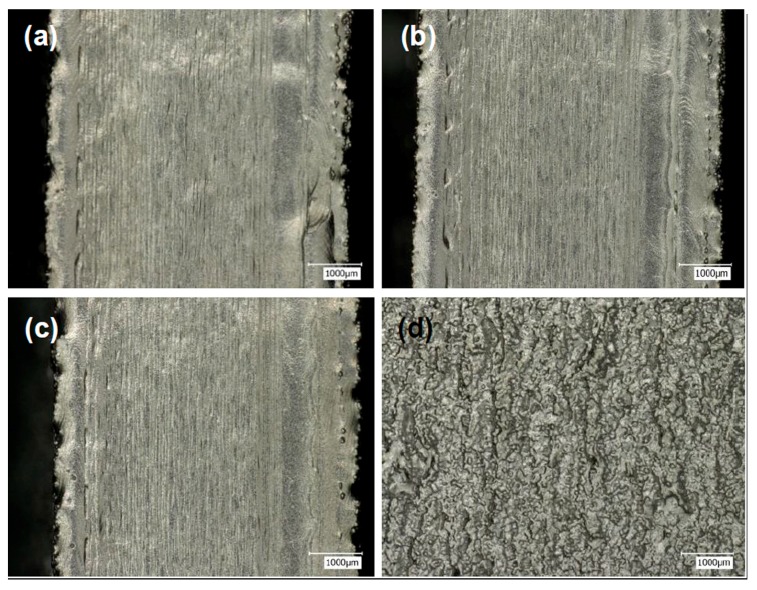
Optical microscopy images showing the surface morphology of (**a**) N1; (**b**) N2; (**c**) N5 from the top view; and the surface conditions of (**d**) N6 from the side view.

The melted outlines formed through the non-multispot contouring were visible from the top of the samples, as shown in Figure 3a–c. They were identical across the experiments except in the thickness of the outlines. Dimension inaccuracies as large as 1 mm were observed as well when measuring the width of the sample using the microscope. This should be considered when the designer prepares the build by applying the non-multispot contouring strategy. From the side view of the experiment N6 (Figure 3d), irregular clustering of melted Ti-6Al-4V was observed, which caused poor surface texture of non-multispot contouring. The reason behind the phenomenon was due to the continuous beam during the contouring process, with heat dissipation mostly downwards to the previously built layers, resulting in the remelting of layers and formation of clusters. The detailed characterization of clusters will be applied by Alicona IFM in the next section.

### 3.2. Multispot Contouring

Table 2 shows the measured surface roughness values in both vertical and horizontal directions for multispot contouring samples. The default processing parameters obtained from Arcam AB (M16) are also listed in Table 2 for control. A surface roughness value of ~31.5 µm in both vertical and horizontal directions was observed in the M16, which agreed well with the previous reports [5,25]. Similar to the results of non-multispot contouring strategy, the surface roughness value of EBM-built sample changes with the modification of the processing parameters in the multispot contouring strategy. It is revealed that number of spots is the dominating factor amongst all the parameters studied. The results from experiment M25 had roughness as low as 27 µm for both the parallel and perpendicular directions, where the maximum number of spots was applied in the present study. In addition, the effects of spot time and spot overlap also contributed to the as-built roughness. Although their effects were not as significant as the number of spots, the best results were achieved by experiments M18, M22, and M25, all of which used 0.4 ms of spot time and 0.6 mm of overlap. The combined effect of the two parameters had a greater influence on the results than each of the parameters on their own. The low spot time and high overlap are the best combination within the current study. On the other hand, beam current and focus offset only exhibited limited effects on the surface roughness. This is likely due to the nature of multispot contouring, the amount of energy transferred from the beam to the powder was relatively small and the effects on the surface roughness had been dominated by other parameters as mentioned earlier.

**Figure 4:**
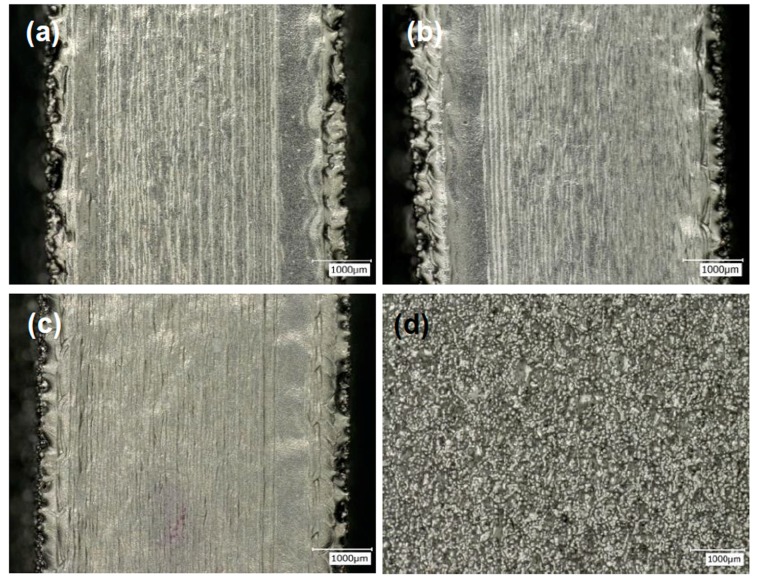
Optical microscopy images showing the surface morphology of (**a**) M3; (**b**) M9; (**c**) M14 from the top view; and the surface conditions of (**d**) M10 from the side view.

Optical microscopic images (Figure 4a–c) did not show any visible outlines which had been observed in the non-multispot counterparts (Figure 3). Instead, the wavy patterns were formed. The phenomenon was caused by the multiple spotting of the beam onto the powder bed during contouring. The outlines were melted instantaneously, but were also allowed time for the melted areas to cool down significantly before the spotting of the adjacent areas along the contour outlines. From the side view of experiment M10, small powder-like features were observed across the samples. The formation of such features was caused by the spotting of the beam. During the contour process, at each instant, the heat from the beam was concentrated on a single spot and this caused the melting of the powder around the region, extending beyond the contour outline. Due to the lack of continuous melting of the outlines and fast cooling of the melted regions, the melted regions beyond the outlines were observed to be smaller than that for the non-multispot samples. Thus ensuring a better dimensional accuracy but with irregular contour outlines.

## 4. Discussion

For non-multispot contouring, the process was optimized with the process parameters of 4 mA for beam current, 4 for speed function, and 0 mA for focus offset. This set of parameters gave the best as-built surface roughness. The results had shown that the surface roughness was the lowest at the mid-point values and this is not in agreement with a previous study [29] which suggested higher scan speed resulting in lower surface roughness. The different versions of Arcam machine, (S-series and A-series) using different control software versions, which may have a variation in scanning strategy, should contribute to the difference. The earlier S-series system had lesser parameters for contour melting as compared to the newer systems such as the A2X [29,31]. It seems that with a higher speed function than 4, the roughness increased and thus followed a nonlinear trend in the present study. As the process requires the electron beam to heat up the powder above the melting point, the contouring process could be viewed as a heat transfer process as well. The heat source (electron beam) needs to have enough energy and time for sufficient heat to be transferred in order for the temperature to reach the melting point of the powder. When the scan speed gets too high, there would have insufficient energy to melt the powder at the contour outline. A similar effect may be observed for the beam current, where the electron beam gets its energy from. A beam current that is too low could result in insufficient energy to melt the powder. Therefore, a rough surface was caused mainly by these defect forms. On the other hand, the opposite end of the values was not ideal as well. If the beam current was too high or the scan speed was too slow, the unstable melting pool caused by over-melting, or even vaporizing of the melted metal, could occur because of too much energy.

For the multispot contouring, the effects of varying spot time had a non-linear trend with the best roughness achieved in the mid-point of the experimental values. The reason was that if the spot time was too long, there would be over-welding of the surrounding powder outside the contour. Conversely, if the spot time was too short, there would be improper melting of the contour. Both instances would result in higher surface roughness. Thus the optimal value for spot time is 0.4 ms. For the effects of the number of spots and the amount of spot overlap, a decreasing roughness with increasing parameter values was observed. A higher number of spots or spot overlap would make the contour melt finer and more uniform, allowing more area along the contour to be melted, thus decreasing the roughness. Hence, for multispot contouring scanning strategy, the process was optimized with the process parameters of >40 for number of spots, 0.4 ms for spot time, 0.6 mm for spot overlap, 3 mA for focus offset, and 4 mA for beam current.

**Figure 5:**
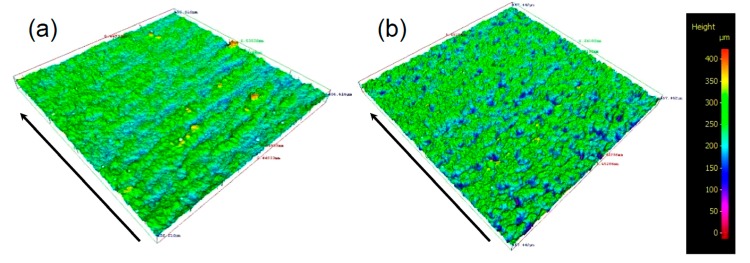
Surface conditions of optimized processing parameters for (**a**) non-mulitspot (N6) and (**b**) mulitspot Ti-6Al-4V sample (M25) observed under Alicona IFM, where the characterized area is 6.5 × 6.5 mm. The arrows indicated the build direction.

It should be noted that the vertical surface roughness values were around 30% higher than the horizontal surface roughness values in non-multispot samples, which was not observed in the multispot samples. To understand this anisotropic surface roughness, the surface conditions of optimized processing parameters for non-mulitspot and mulitspot Ti-6Al-4V samples were observed under the Alicona IFM, as shown in Figure 5. The arrow indicated the build direction. Both of them exhibited peaks and valleys. The peaks (also indicated as clusters under an optical microscope, as shown in Figure 3d) for the multispot sample were more uniform than the non-multispot sample. However, some layer-wise features were observed in the non-multispot sample (Figure 5a) which may be caused by the unstable melting pool, as illustrated in Figure 2a. It is clearly seen that the multispot sample gave a more uniform surface than the non-multispot.

Although the surface roughness value becomes low in the optimized processing parameters (N6 in Table 1 and M25 in Table 2) comparing to the default processing parameters (M16 in Table 2), the window for improvement by modifying only the processing parameters is limited. By applying finer powder and thinner layer thickness, the surface quality is expected to be further improved, which may reduce the post treatments. However, the finer powder is expected to be expensive which increases the overall fabrication cost. The thinner layer thickness increases the processing time, in turn, decreases the productivity and increases the fabrication cost. Therefore, a trade-off between the EBM processing cost and post treatments should be considered. The improved surface quality in the present study should make the mechanical response better, especially for the thin wall structure and small struts. Although no study has been reported yet and this hypothesis should be further investigated, previous studies [3,24] have shown that improved surface quality by chemical etching enhances the mechanical response of EBM-built tensile specimens and lattice structures. The mechanical response of thin wall samples with varying processing parameters should be further investigated in the futures.

**Figure 6:**
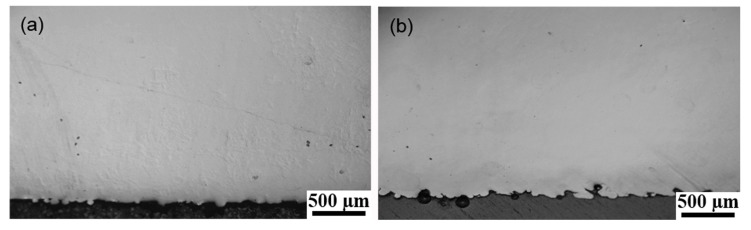
Optical microscope images showing the cross sections of (**a**) N6 and (**b**) M22.

For the production, the fusion condition between the contouring and hatching also should be considered, especially for the optimized processing parameters that would be applied in the near future. Therefore, the cross-section views of N6 and M25 were observed by optical microscope, as shown in Figure 6. There were no defects found between the contouring and hatching interface. Thus, both the non-multispot and multispot contouring scanning strategies could be used to build parts without compromising the mechanical properties. In addition, the dimensional accuracy seems to be compromised by the reduced roughness, when comparing between non-multispot and multispot contouring strategies. The build time for the non-multispot contouring scanning strategy was also longer than the multispot contouring scanning strategy. Thus, the choice of which contouring method should be used would depend on the intended applications and the geometric dimensioning specifications for the built part. When it comes to industrial applications for EBM-built parts, the dimensional accuracies and as-built surface roughness should be taken into consideration. If the manufacturer wishes to have a better as-built dimensional accuracy and/or post-processes planned for the part, the build could be built using the multispot contouring scanning strategy. Conversely, the manufacturer wishes to have a better as-built surface finish without concerns of dimensional accuracy and/or build time, the build could be built using the non-multispot contouring scanning strategy.

## 5. Conclusions

The effects of processing parameters on the part’s surface roughness were systemically studied. Based on the results, the following conclusions can be drawn:

(1) The non-multispot contouring strategy produced lower as-built surface roughness but compromised dimensional accuracy while the multispot contouring strategy had higher as-built roughness but better dimensional accuracy. Moreover, an anisotropic surface roughness was observed in the sample with non-multispot contouring strategy, which was caused by the unstable melting pool. The vertical surface roughness values were around 30% higher than the horizontal surface roughness values in non-multispot samples.
(2) For the non-multispot contouring scanning strategy, lower beam current and speed function resulted in better as-built surface roughness. The vertical and horizontal surface roughness values of optimized conditions were 24 µm and 20 µm, respectively.
(3) For the multispot contouring scanning strategy, high number of spots, with a spot time of 0.4 ms, and high spot overlap had contributed to better as-built part’s roughness. Amongst all of the tested processing parameters, number of spots was the dominating factor. The results from the optimized sample, M25, were ~27 µm for both the vertical roughness and horizontal roughness.

## Figures and Tables

**Table 1:** Surface roughness results of non-multispot contouring. All the average values and standard deviations were obtained by measuring at least five values.

	Beam Current (mA)	Speed Function	Focus Offset (mA)	Vertical Surface Roughness (µm)	Horizontal Surface Roughness (µm)
**N1**	2	2	3	33.6 ± 2.1	22.8 ± 1.9
**N2**	2	4	3	29.3 ± 1.3	23.1 ± 2.2
**N3**	2	6	3	33.3 ± 1.3	23.6 ± 1.1
**N4**	4	2	3	28.1 ± 1.8	21.6 ± 1.1
**N5**	4	4	0	24.1 ± 2.3	19.7 ± 1.3
**N6**	4	4	3	25.4 ± 2.1	21.7 ± 0.7
**N7**	4	6	0	33.5 ± 6.5	25.1 ± 3.3
**N8**	4	6	3	32.1 ± 4.6	31.2 ± 4.9
**N9**	6	4	0	31.0 ± 5.8	24.9 ± 5.2
**N10**	6	4	3	33.0 ± 4.9	24.3 ± 4.7
**N11**	6	6	0	33.8 ± 3.3	24.3 ± 2.7
**N12**	6	6	3	39.3 ± 6.7	30.9 ± 2.8

**Table 2:** Surface roughness results of multispot contouring. All the average values and standard deviations were obtained by measuring at least five values.

	Number of Spots	Spot Time (ms)	Spot Overlap (mm)	Focus Offset (mA)	Beam Current (mA)	Vertical Surface Roughness (µm)	Horizontal Surface Roughness (µm)
**M1**	10	0.6	0.2	1	4	40.5 ± 2.7	32.3 ± 2.3
**M2**	10	0.6	0.2	1	6	37.7 ± 2.9	29.2 ± 2.8
**M3**	10	0.6	0.2	3	4	38.2 ± 2.1	31.4 ± 2.9
**M4**	10	0.6	0.2	3	6	37.3 ± 3.5	28.4 ± 3.9
**M5**	10	0.8	0.4	3	4	32.6 ± 1.7	30.0 ± 1.4
**M6**	40	0.6	0.2	1	4	31.8 ± 1.9	31.5 ± 2.1
**M7**	40	0.6	0.2	1	6	31.8 ± 2.6	30.5 ± 2.9
**M8**	40	0.6	0.2	3	4	29.6 ± 2.3	28.9 ± 2.2
**M9**	40	0.6	0.2	3	6	33.6 ± 1.6	31.4 ± 1.3
**M10**	40	0.6	0.4	1	6	29.8 ± 1.5	29.7 ± 3.7
**M11**	40	0.8	0.2	1	6	33.0 ± 1.2	30.3 ± 1.4
**M12**	40	0.8	0.2	3	6	30.7 ± 1.4	29.1 ± 2.4
**M13**	40	0.8	0.4	1	4	31.6 ± 1.4	30.5 ± 2.0
**M14**	40	0.8	0.4	1	6	34.7 ± 3.1	33.0 ± 1.7
**M15**	40	0.8	0.4	3	4	30.9 ± 1.2	30.6 ± 2.0
M16 *	40	0.8	0.2	3	4	31.7 ± 1.9	31.0 ± 1.9
**M17**	55	0.4	0.4	3	4	27.3 ± 1.7	25.5 ± 3.2
**M18**	55	0.4	0.6	3	4	26.1 ± 0.9	27.0 ± 2.9
**M19**	55	0.8	0.4	3	4	32.4 ± 1.3	31.5 ± 2.1
**M20**	55	0.8	0.6	3	4	32.9 ± 1.3	30.6 ± 2.4
**M21**	70	0.4	0.4	3	4	28.9 ± 2.0	27.8 ± 1.9
**M22**	70	0.4	0.6	3	4	28.4 ± 1.2	25.8 ± 1.9
**M23**	70	0.8	0.4	3	4	35.1 ± 3.8	33.3 ± 2.3
**M24**	70	0.8	0.6	3	4	33.6 ± 1.9	32.3 ± 1.6
**M25**	80	0.4	0.4	3	4	27.9 ± 1.4	27.1 ± 1.9
**M26**	80	0.4	0.6	3	4	29.3 ± 1.7	28.0 ± 2.5
**M27**	80	0.8	0.4	3	4	34.2 ± 1.7	32.6 ± 2.0
**M28**	80	0.8	0.6	3	4	34.7 ± 1.5	33.5 ± 1.9

* The default processing parameters obtained from Arcam AB.
